# Oncolytic Viruses for Cancer Therapy: Overcoming the Obstacles

**DOI:** 10.3390/v2010078

**Published:** 2010-01-11

**Authors:** Han Hsi Wong, Nicholas R. Lemoine, Yaohe Wang

**Affiliations:** 1 Centre for Molecular Oncology and Imaging, Institute of Cancer, Barts and The London School of Medicine and Dentistry, Queen Mary University of London, London EC1M 6BQ, UK; E-Mails: h.h.wong@qmul.ac.uk (H.H.W.); director@qmcr.qmul.ac.uk (N.R.L.); 2 Sino-British Research Centre for Molecular Oncology, Zhengzhou University, Zhengzhou 450052, China

**Keywords:** oncolytic virus, adenovirus, vaccinia virus, cancer gene, host immune response

## Abstract

Targeted therapy of cancer using oncolytic viruses has generated much interest over the past few years in the light of the limited efficacy and side effects of standard cancer therapeutics for advanced disease. In 2006, the world witnessed the first government-approved oncolytic virus for the treatment of head and neck cancer. It has been known for many years that viruses have the ability to replicate in and lyse cancer cells. Although encouraging results have been demonstrated *in vitro* and in animal models, most oncolytic viruses have failed to impress in the clinical setting. The explanation is multifactorial, determined by the complex interactions between the tumor and its microenvironment, the virus, and the host immune response. This review focuses on discussion of the obstacles that oncolytic virotherapy faces and recent advances made to overcome them, with particular reference to adenoviruses.

## 1. Introduction

Cancer is a major cause of death globally. Although treatments for the disease have improved significantly, conventional chemotherapy or radiotherapy still have limited effects against many forms of cancer, not to mention a plethora of treatment-related side effects. This situation signifies a need for novel therapeutic strategies, and one such approach is the use of viruses. The ability of viruses to kill cancer cells has been recognized for more than a century [1]. They achieve this by a number of mechanisms, including direct lysis, apoptosis, expression of toxic proteins, autophagy and shut-down of protein synthesis, as well as the induction of anti-tumoral immunity. Although clinical trials of several naturally-occurring oncolytic viruses were started back in the 1950s, it was only in 1991 that a herpes simplex virus-1 (HSV-1) with deletion of its thymidine kinase *UL23* gene became the first genetically-engineered, replication-selective oncolytic virus to be tested in the laboratory [2]. In 2005, an adenovirus (Ad) with *E1B 55K* gene deletion (H101(Oncorine); Shanghai Sunway Biotech, Shanghai, China) was approved in China as the world’s first oncolytic virus for head and neck cancer in combination with chemotherapy [3]. However, until now the widespread use of oncolytic virotherapy is still far from reality. Promising laboratory results have not been translated to improved clinical outcomes, and this appears to be determined by the complex interactions between the tumor and its microenvironment, the virus, and the host immunity. There are already a number of reviews on oncolytic viruses for cancer treatment but this article will focus on the obstacles facing oncolytic virotherapy, with particular reference to Ads, and the recent advances made to overcome these hurdles.

### Mechanisms of tumor selectivity

**Figure 1.**
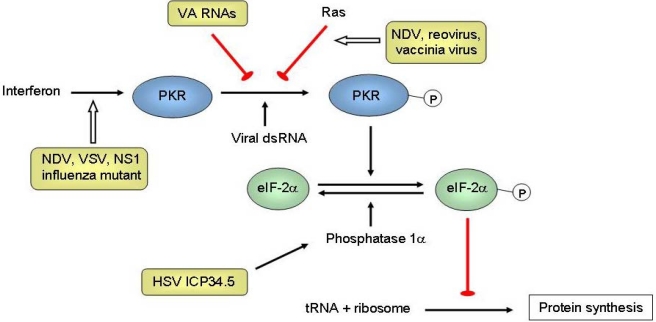
Mechanisms of tumor selectivity of several oncolytic viruses. The interferon (IFN)/double-stranded RNA-activated protein kinase (PKR) pathway is a natural anti-viral defense system. IFNs produced by infected cells result in the upregulation of PKR. On binding to viral double-stranded RNA (dsRNA), PKR autophosphorylates, which in turn phosphorylates the α subunit of eIF-2. Phosphorylated eIF-2α sequesters eIF-2B, a guanine nucleotide exchange factor. Without eIF-2B, the GDP bound to eIF-2 cannot be exchanged for GTP. As a result eIF-2 is unable to bring the initiator transfer RNA (tRNA) to the 40S ribosomal subunit, and the synthesis of viral protein is inhibited. Inactivated IFN and activated Ras pathways are frequently found in cancer (the latter could inhibit PKR), and some naturally-found viruses can replicate selectively in cancer but not normal cells, including the Newcastle disease virus (NDV) [21], reovirus [22], vaccinia virus [23], and vesicular stomatitis virus (VSV) [24]. The herpes simplex virus (HSV) protein ICP34.5 interacts with cellular phosphatase 1α to dephosphorylate eIF-2α, leading to synthesis of proteins needed for virus replication. Deletion of gene that encodes for ICP34.5 (*RL1*) results in selective replication in tumors with a defective IFN/PKR pathway [25]. The influenza virus *NS1*-deleted mutant is also dependent on this defective pathway [26]. Adenoviruses normally produce virus-associated (VA) RNAs to inhibit PKR. As such, engineered *VAI*-deleted adenovirus (*dl*331) could replicate selectively in tumors with an activated Ras pathway [27]. Epstein-Barr virus (EBV) also expresses RNAs similar to VA RNAs and these can complement *dl*331, resulting in selectivity in EBV-associated tumors [28].

**Figure 2.**
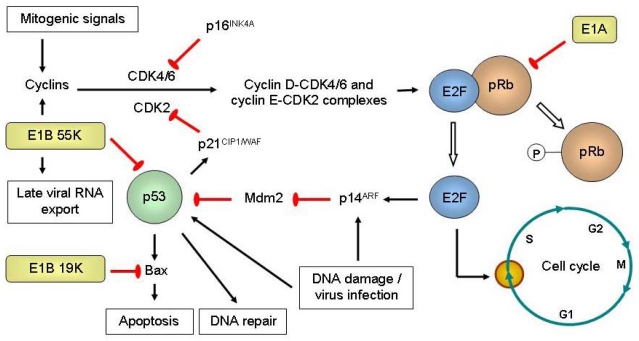
Engineered replication selectivity of oncolytic adenoviruses (Ads) by deletion of the *E1A*, *E1B 19K* or *E1B 55K* gene. Retinoblastoma protein (pRb) is normally hypophosphorylated and binds to transcription factors of the E2F family to regulate the G1-to-S checkpoint of the cell cycle. Upon stimulation by mitogenic signals, upregulation of cyclins enables cyclin-dependent kinases (CDKs) to phosphorylate pRb, releasing E2F that leads to the expression proteins needed for DNA synthesis and thus cell cycle progression. E2F upregulates p14^ARF^, which inhibits Mdm2. Mdm2 normally results in p53 degradation. p53 is a transcription factor that is upregulated and activated by stress signals such as virus infection or DNA damage. It results in the expression of proteins that induce apoptosis (Bax), cell cycle arrest (p21^CIP1/WAF^ via its inhibition of CDK2) or DNA repair. p16^INK4A^ is a tumor suppressor that inactivates CDK4/6. The adenoviral E1A proteins bind to pRb to release E2F, so that viral DNA could be replicated. E1A also promotes the acetylation of pRb by p300/CBP, causing pRb to associate with Mdm2 to inhibit p53. Because cancer cells are often in the S phase, *E1A CR2*-deleted Ad5 mutant (*dl*922-947) could selectively replicate in and destroy replicating cancer cells but not normal resting cells [29]. E1B 19K binds to and inhibits Bax. The tumor selectivity of *E1B 19K*-deleted Ad2 (*dl*250) is due to multiple defects in the apoptotic pathways, where survival of the virus in normal cells would be limited owing to rapid apoptosis induction in the presence of tumor necrosis factor-α (TNF-α) [30]. E1B 55K interacts with the adenovirus E4 open reading frame 6 (E4orf6) protein to form an E3 ubiquitin ligase complex that targets p53 for degradation. It also induces the expression of cyclin E as well as simultaneously inhibits cellular mRNA export and promotes the export of late viral mRNAs. *E1B 55K*-deleted Ad could replicate in tumor selectively because of non-functioning p53 [31], cyclin E overexpression [32], and E1B 55K-independent late viral RNA export in cancer but not normal cells [33].

The term ‘oncolytic viruses’ applies to viruses that are able to replicate specifically in and destroy tumor cells, and this property is either inherent or genetically-engineered. Inherently tumor-selective viruses can specifically target cancer by exploiting the very same cellular aberrations that occur in these cells, such as surface attachment receptors, activated Ras and Akt, and the defective interferon (IFN) pathway (Figure 1). Some viruses have been engineered with specific gene deletion – these genes are crucial for the survival of viruses in normal cells but expendable in cancer cells (Figure 2). Deletion of the gene that encodes thymidine kinase, an enzyme needed for nucleic acid metabolism, results in dependence of viruses such as HSV and vaccinia virus on cellular thymidine kinase expression, which is high in proliferating cancer cells but not in normal cells. Vaccinia also produces the vaccinia growth factor (VGF) that binds to and activates the epidermal growth factor receptor (EGFR), creating an environment that supports its replication. It follows that deletion of genes encoding for both thymidine kinase and VGF leads to further selectivity of vaccinia virus in cancers with an activated EGFR-Ras pathway [4]. Another approach in conferring tumor selectivity is to restrict virus replication by its dependence on transcriptional activities that are constitutively activated in tumor cells. This can be achieved by the insertion of a tumor-specific promoter driving the expression of a critical gene [5–11]. Others viruses either possess naturally (e.g., Coxsackievirus A21 [12] and measles virus (MV) [13]) or have been designed to have specific tropism based on the expression of cell surface receptors unique to cancer cells [14–20].

More recently, gene silencing by RNA interference technology has been utilized to confer tumor selectivity. MicroRNAs (miRNAs) or small interfering RNAs (siRNAs) regulate gene expression post-transcriptionally by translation block or cleavage of specific, complementary mRNA via the RNA-induced silencing complex (RISC). By inserting a complementary sequence next to a critical viral gene, it is possible to confine virus replication to tumor but not normal cells that express high levels of the corresponding miRNA. This has been demonstrated by several groups [34–38]. Gürlevik *et al.* [39] developed a recombinant Ad that encodes multiple RNA-interfering transcripts under the control of a p53-responsive promoter. The transcripts could effectively silence a set of critical viral genes. As p53 is a transcription factor often lost or mutated in human malignancy, this virus could therefore replicate in cancer but not normal cells where functional p53 would lead to an anti-viral RNA interference.

### Optimizing oncolytic viruses for improved anti-tumoral potency

Gene-manipulated oncolytic viruses such as Ad, herpes virus and vaccinia virus are being developed as a new class of anti-tumoral agent [23,40,41]. Selective intratumoral replication of the virus may lead to improved efficacy over non-replicating agents due to the self-perpetuating nature of the treatment with virus multiplication, lysis of the infected tumor and spread to adjacent cells. One potential limitation of this approach, however, is that gene deletions resulting in tumor selectivity also frequently result in reduced oncolytic potency. For example, *dl*1520 (ONYX-015; Onyx Pharmaceuticals, California, USA) is an oncolytic Ad2/Ad5 hybrid with deletion of its *E1B 55K* and *E3B* genes. The E1B 55K protein is involved in p53 inhibition, viral mRNA transport and host cell protein synthesis shut-off [42] (Figure 2), whilst E3B proteins are important for immune avoidance (see below). This virus was the first engineered, replicating Ad to enter clinical trials for cancers including those of the head and neck [43–45] and pancreas [46,47]. Whilst the virus has shown good tumor selectivity and safety [48], durable objective responses with this virus as a single agent have been limited and this could be partly due to the loss of other essential functions of the *E1B 55K* and *E3B* genes. A recent finding by Thomas *et al*. [49] revealed that *dl*1520 was less efficient in lysing cells infected in the G1 phase of the cell cycle due to a reduced rate of late viral protein synthesis, and this appears to be a result of the adenoviral gene product encoded by open reading frame 1 of early region 4 (*E4orf1*). As such there is a need to increase the potency of these viruses by identifying mutations that result in tumor selectivity but not those that result in attenuated virus replication and oncolysis. Since the first generation of replication-selective Ads was tested in pre-clinical experiments and clinical trials, several advances have been made to improve potency by dissecting the functions of different genes of Ad.

The adenoviral *E1A* is the earliest gene to be transcribed after virus entry into the host cell [50]. E1A normally interacts with the retinoblastoma protein (pRb) (the latter is important in regulating the G1-to-S cell-cycle checkpoint), and this pushes quiescent cells into S phase to allow for virus replication (Figure 2). Therefore, *dl*922–947, the mutant Ad with specific deletion of the *E1A CR2* region (pRb binding site), was unable to replicate in quiescent normal cells but was able to do so in cancer cells with defective G1-to-S checkpoint. This virus has demonstrated superior anti-tumoral activity *in vivo* compared to *dl*1520 after intratumoral and intravenous injections [29], although it might also target proliferating non-malignant cells. In addition to its effect on virus release and spread [51,52], adenoviral E1B 19K is a functional homolog of Bcl-2 and is able to bind to Bax [53–55] and also prevent Fas-mediated apoptosis [56]. Replication of the mutant Ad2 with *E1B 19K* deletion (*dl*250) was significantly reduced in normal cells secondary to rapid apoptosis induction in the presence of tumor necrosis factor-α (TNF-α), whilst the opposite occurred in cancer cells due to multiple defects in the apoptotic pathways (e.g., p53 mutation, Bcl-2 overexpression) [30] (Figure 2). Virus replication, spread and anti-tumoral potency was significantly better than *dl*1520 and wild-type Ad2. *E1B 19K*-deleted Ad5-infected cancer cells also expressed lower levels of EGFR and anti-apoptotic proteins [57].

Ads also produce the virus-associated (VA) RNAs. These are RNA polymerase III transcripts that, amongst other functions, are obligatory for efficient translation of viral and cellular mRNAs by blocking the double-stranded RNA-activated protein kinase (PKR) [58,59], a natural host anti-viral defense system (Figure 1). We have shown that *VAI*-deleted Ad5 (*dl*331) was able to selectively target Epstein-Barr virus (EBV)-associated tumors such as Burkitt’s lymphoma and nasopharyngeal carcinoma [28]. This is because EBV expresses the RNAs EBER1 and EBER2, whereby EBER1 could complement *dl*331 to enable the synthesis of viral proteins. Interestingly, anti-tumoral efficacy *in vitro* and *in vivo* was superior to wild-type Ad5 and this might be the result of PKR-induced apoptosis, increased IFN-β production, and the adenoviral *E3B* gene deletion.

Gene products encoded by the adenoviral *E3* region could also affect its oncolytic potency. These include the *E3 11.6K* (or adenovirus death protein – ADP), which facilitates late cytolysis of infected cells and release of progeny viruses [60]. Ads that overexpress ADP showed better cell lysis and spread [61,62]. The effects of *E3B* and *E3 gp19K* genes on the potency of oncolytic adenovirus will be discussed later.

### Arming oncolytic viruses with therapeutic genes

The discovery of the genetic basis of malignancy has in part promoted the development of cancer gene therapy, which involves the introduction of exogenous nucleic acid to restore, express or inhibit a particular gene of interest. Viruses are at present the most efficient gene delivery system. A well-known example is Gendicine (Shenzhen SiBiono GeneTech, Shenzhen, China), an Ad5 vector encoding the human *TP53* gene that was approved in 2004 by China’s State Food and Drug Administration for the treatment of head and neck cancer [63]. Although developed for safety reasons, one major shortcoming of using non-replicating vectors such as Gendicine (by virtue of its *E1A* gene deletion) is that infectivity is limited to only one cycle. In contrast, oncolytic viruses can replicate and spread in cancer cells resulting in longer transgene expression. Together with tumor lysis this would lead to better therapeutic efficacy. Arming oncolytic viruses with anti-cancer genes has been a major focus in cancer virotherapy, and transgenes exploited include tumor suppressor, pro-apoptotic, anti-angiogenic, “suicide”, and immunomodulatory genes.

Like Gendicine, oncolytic viruses could be armed with tumor suppressor or pro-apoptotic genes that are frequently lost in cancer. One example is by the use of p16^INK4A^-armed oncolytic Ad, which has shown good inhibition of gastric tumor xenografts [64]. Wang *et al*. [65] developed an Ad in which the *E1A* gene is regulated by the human telomerase reverse transcriptase (hTERT) promoter and hypoxia response element, together with p53 under the strong cytomegalovirus (CMV) promoter. This virus showed tumor selectivity with efficient p53 expression and oncolysis. Nonetheless, targeting a single gene is unlikely to have a major impact on survival, given that in cancer a large number of genetic alterations affect only a core set of signaling pathways and processes, as has been recently described for pancreatic cancer [66]. Hence there should be a move from targeting these genes individually to targeting cancer signaling pathways, such as arming oncolytic Ad with an engineered transgene that encodes transforming growth factor (TGF)-β receptor II fused with the human Fc IgG_1_, as studied by Hu *et al*. [67]. Anti-tumoral effects were observed with a replication-selective (but not replication-deficient) virus encoding this gene, highlighting the importance of virus replication. Viruses that enhance the apoptotic pathways have also been studied. Jin *et al*. [68] and Chen *et al*. [69] utilized the chimeric Ad5/35 carrying the gene encoding the TNF-related apoptosis-inducing ligand (TRAIL) to promote receptor-independent infection (see below) and apoptosis of leukemic and gastric cancer cells, respectively. Zhang *et al*. [70] treated pancreatic cancer cells by replacing the gene for human somatostatin receptor 2 (lost in 90% of pancreatic cancers) and introducing the gene for TRAIL by means of an oncolytic Ad, with good results *in vivo*. A reciprocal approach is to ablate the function of oncogenes post-transcriptionally by arming oncolytic Ad with small hairpin RNA (shRNA). Recent work includes those targeting hTERT [71], Ki-67 [72], Survivin [73], and Apollon [74], all of which have shown efficient anti-tumoral effects *in vitro* and *in vivo*.

The tumor microenvironment plays a critical role in promoting malignant cell growth and progression, as well as restricting virus spread. One important issue is tumor angiogenesis. A recent finding by Ikeda *et al*. [75] suggested that the replication-selective Ad OBP-301, in which the *E1* genes are under the control of the hTERT promoter, could stimulate peripheral blood mononuclear cells (PBMCs) to produce IFN-γ that has anti-angiogenic properties, resulting in reduced tumor vascularity and slowed growth in immunocompetent mice. However, Kurozumi *et al*. [76] also showed that intratumoral treatment of rat glioma with oncolytic HSV could promote neovascularization of the residual tumor, and this was associated with a significant increase in the angiogenic factor CYR61. This could have an impact on subsequent tumor growth and the observation suggests that a combination of oncolytic virus with anti-angiogenic transgene might be needed; for this we refer the reader to our recent article for a more comprehensive review [77]. Recent work includes the use of the anti-angiogenic factors endostatin/angiostatin [78–80], interleukin-18 (IL-18) [81,82], canstatin [83], and trichostatin A [84], as well as arming viruses with genes that inhibit pro-angiogenic molecules such as IL-8 [85] and vascular endothelial growth factor (VEGF) [86,87]. Kang *et al*. [88] made use of a transcriptional repressor based on zinc-finger protein to target the VEGF promoter. An oncolytic Ad armed with this gene significantly reduced vessel density and tumor size of human glioblastoma xenografts in mice. The matrix metalloproteinases (MMPs) are a family of proteolytic enzymes that degrade the extracellular matrix and are essential for tumor spread and neovascularization. Oncolytic viruses armed with genes that encode MMP inhibitors have shown encouraging results in delaying tumor growth and angiogenesis [89,90].

Gene-directed prodrug activation therapy (or suicide gene therapy) involves the delivery of a gene that would lead to the expression of an enzyme, followed by the administration of a prodrug that is activated selectively by this enzyme. One example is the HSV thymidine kinase (HSV-TK)-ganciclovir method, whereby HSK-TV is able to monophosphorylate ganciclovir, which is subsequently converted by cellular kinases to the triphosphorylated forms, blocking DNA synthesis and inducing cell death. Most publications have described the use of replication-deficient viruses with this approach, but recent studies that demonstrated its efficacy using replication-selective oncolytic Ads include treatment for prostate [91], gallbladder [92], and liver [93] cancers. Alternative combinations include nitroreductase with the prodrug CB1954 (converted into an alkylating agent) [94], and cytosine deaminase (CD) with 5-fluorocytosine, which is converted into the cytotoxic and radiosensitizing 5-fluorouracil [95,96]. An Ad5 with *E1B 55K* deletion, ADP overexpression and CD/TK fusion gene expression is currently in a phase III trial in combination with radiotherapy for patients with prostate cancer.

### The tumor environment and oncolytic viruses

Viruses are naturally larger than other anti-cancer agents such as chemicals and antibodies (for example 90 nm and 300 nm for Ad and vaccinia virus, respectively). After intratumoral injection, effective virus spread could be impaired by the extracellular matrix, areas of fibrosis and necrosis, and surrounding normal cells in the tumor bed, although Kolodkin-Gal *et al*. [97] found that the extracellular components collagen and mucin could restrict HSV-1 infectivity in normal colon, but these molecules were expressed in lesser amounts in colonic carcinoma, facilitating its spread. Ganesh *et al*. [98] studied the co-administration of the enzyme hyaluronidase with oncolytic Ads during intratumoral injection. This degraded the major constituents of the extracellular matrix, hyaluronan, resulting in enhanced virus spread *in vivo*. Induction of cancer cell death with an apoptosis-inducing agent prior to injection of oncolytic HSV could also produce channels for effective virus spread [99]. Elevated interstitial hydrostatic pressure as a result of fibrosis and vessel abnormalities poses another physical barrier to successful virus delivery and this effect increases with tumor volume [100]. Injected viruses could escape back through the injection site or by drainage into the circulation, resulting in reduced efficacy and increased risk of systemic toxicities. Bazan-Peregrino *et al.* [101] examined the retention of Ad5 in MDA-231 and ZR75.1 human breast carcinoma xenografts after intratumoral injection. For MDA-231, occlusion of injection sites with surgical adhesives and the use of small injected volumes resulted in significantly higher virus retention within the tumors. ZR75.1, however, took up more Ad than MDA-231 when identically infected, suggesting a role of tumor type in virus retention. Recently, tumor-associated stromal cells have been shown to play a role in either enhancing or reducing the efficacy of oncolytic Ads, depending on the tumor type [102]. Hypoxia, a common feature in tumor tissues, has been found to reduce the replicative and oncolytic potential of Ads despite the unaltered expression of surface receptors [103,104]. In this regard there might be a role for the development of oncolytic viruses in which replication is not attenuated by hypoxia, such as vaccinia virus [105] or HSV [106,107].

For viruses that have reached the immediate vicinity of the tumor, cellular genetic changes could prevent successful virus entry into the cells. For cellular entry of most Ads (those in subgroups A, C, D, E and F – which include the commonly used Ad5), they must first bind to the Coxsackie and adenovirus receptor (CAR) on the surface membrane via the knob portions of their fibers, followed by internalization mediated by the viral penton proteins and cellular integrins. CAR is ubiquitously expressed in epithelial cells, but its expression is often downregulated in many cancer types due to activation of the Raf-MAPK pathway [108]. Recent work has shown that the molecule leucine-rich repeat-containing protein 15 (LRRC15 or hLib), frequently overexpressed in tumor cells, could result in the redistribution of CAR away from cell surfaces, thus impeding Ad infection [109]. In contrast, most subgroup B Ads bind to CD46 [110], a receptor often upregulated in a number of tumor types, including breast, cervical, liver, lung, endometrial and hematological malignancies [111–113]. Several chimeric oncolytic Ad5 have been developed to contain the fiber tropism of subgroup B Ads and they all have shown encouraging results [68,69,114–117]. The use of intact subgroup B Ads as oncolytic agents is still under-explored but has great potential [118,119]. They have different tropism and infectivity compared to chimeric viruses [120], and are more beneficial in terms of a reduced propensity for neutralization by pre-existing antibodies (see below). Besides CD46, evidence suggests that the subgroup B Ad, Ad11, also utilizes another unidentified receptor [121,122], tentatively named ‘receptor X’ by Tuve *et al*. [123]. They also discovered that the other subgroup B Ads, Ad16, -21, -35 and -50 exclusively use CD46, whereas Ad3, -7 and -14 use ‘receptor X’ but not CD46. It is possible that Ad11 could infect a wider range of tumor cells and overcome receptor downregulation; the latter is a known problem with Ad35 and CD46 [124]. Strauss *et al*. [125] showed that Ads that utilize CAR or CD46 as primary attachment receptors failed to infect and lyse ovarian cancer cells of the epithelial phenotype, which are found in *in situ* tumors and tumor xenografts. These receptors are trapped in the tight junctions and therefore not accessible to the virus. However, Ads that use receptor X (Ad3, -7, -11 and -14) could induce epithelial-mesenchymal transition and result in efficient oncolysis.

Cellular signaling pathways can also affect virus infectivity. Recently our group [126] has shown that certain pancreatic cancer cell lines overexpress the carcinoembryonic antigen–related cell adhesion molecule 6 (CEACAM6), which antagonizes the Src signaling pathway, downregulates cancer cell cytoskeleton proteins, and blocks Ad trafficking to the nucleus. Knockdown of CEACAM6 by siRNA significantly enhanced the anti-tumoral potency of oncolytic Ad5. For virus that has successfully entered the cell, it needs to replicate for efficient cell lysis and virus spread. The protein p21^CIP1/WAF^ normally inhibits cyclin-dependent kinase 2 (CDK2) (Figure 2) and blocks the progression of the cell cycle from G1 to S phase. Shiina *et al*. [127] showed that siRNA knockdown of p21^CIP1/WAF^ increased Ad replication and oncolysis. It was suggested that this could be due to the inhibition of SET and proliferating cell nuclear antigen (PCNA) by p21^CIP1/WAF^, whereby SET and PCNA normally increase viral DNA replication. In the case of vaccinia virus, recent work has suggested that cells with activated c-Jun NH2-terminal kinase (JNK) signaling cascade could activate PKR (Figure 1), thus reducing virus replication [128].

Cancer stem cells form part of the heterogenous tumor population. They not only contribute to neoplastic progression and metastasis, but also to resistance to chemotherapy and radiotherapy. Evidence has shown that oncolytic Ads are able to destroy these cells [129–131]. Zhang *et al*. [132] have recently demonstrated that a telomerase-specific oncolytic Ad armed with a gene that encodes the apoptotic TRAIL was able to preferentially target stem-like esophageal cancer cells and prolong the survival of mice bearing tumors composed of these cells. Whilst this is of interest, cancer stem cells only form a small subset of the tumor mass and the value of targeting them specifically will remain an issue to be resolved.

### Modification of the host immune response in favor of oncolytic viruses

Most studies of oncolytic viruses have been done, by necessity, on human tumor xenografts in immunodeficient mice – far from reflective of the human condition. Unsurprisingly, data from these studies have not been predictive for clinical trial results. The effects of the host immune response on the efficacy of oncolytic viruses are complex. When stimulated, immune cells could result in virus clearance but might also induce specific and non-specific anti-tumoral activities. It appears that the innate immune response plays an important role in virus clearance, whereas T cell-mediated responses are largely responsible for the anti-tumoral effect [133–137].

**Figure 3.**
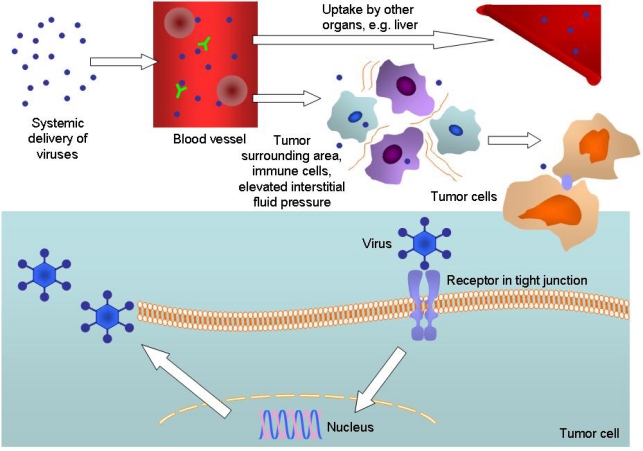
Obstacles to successful delivery of oncolytic viruses to tumor cells. After intravenous injection, viruses are neutralized by pre-existing antibodies and complement activation. Adenoviruses (Ads) also interact with blood cells. Recent work has revealed that Ad5 binds to erythrocytes via the Coxsackie and adenovirus receptor (CAR) and complement receptor 1 (CR1) in the absence and presence of anti-Ad5 antibodies, respectively [178]. Sequestration into other organs and the reticuloendothelial system is a particular problem, often with resulting toxicities. From the blood stream, viruses have to pass through a mixture of extracellular matrix, cells (including normal and immune cells) and high interstitial fluid pressure before reaching the tumor. They then have to attach to the cellular receptor (often trapped in tight junction), be internalized, translocate to the nucleus, replicate, produce structural and other proteins, lyse the cell and release their progenies – some of these steps could be inhibited by factors such as the natural host immune response, hypoxic environment, soluble factors, and genetic changes in the tumor cell.

For the treatment of metastatic or hematological malignancies, intravenous virus delivery could be hindered by neutralizing antibodies, complement activation, non-specific uptake by other tissues such as the liver and spleen, as well as poor virus escape from the vascular compartment (Figure 3). For Ad, adhesion to blood cells could also lead to therapeutic inhibition [138]. Numerous experiments have been done to modify the immune response in favor of virus replication and tumor lysis. One method is by using an immunosuppressive agent, such as cyclophosphamide, that has been shown to improve virus spread and anti-tumoral efficacy [139–145]. Kurozumi *et al*. [146] found that single doses of the angiostatic and anti-inflammatory cyclic peptide of arginine-glycine-aspartic (cRGD), given before an oncolytic HSV, resulted in reduced tumor vessel permeability, leukocyte infiltration and IFN-γ, leading to increased survival of rats with intracranial gliomas. Various data suggest that pre-existing antibodies decrease virus spread after intravenous delivery [147–149], but have a lesser effect on intratumoral injection [44,150,151]. Although antibodies could prevent possible toxicity [152], they could also reduce efficacy. Possible ways to circumvent this include plasmapheresis to deplete antibodies and the use of other viral strains with a lower prevalence of antibodies in the human population. One example is Ad11 [118,119], with a reported antibody prevalence of 10–31% compared to 45–90% for Ad5 [122,153–155]. These antibodies are mainly directed against the viral hexon proteins [156], suggesting that the use of Ad11 virion might be better than chimeric Ad5/11, where the fibers are derived from Ad11 but the rest, including hexon, belong to Ad5. A caveat to this is that for unknown reasons, Ad11 appears to induce more pro-inflammatory cytokines and chemokines than Ad5 or Ad5/11 in mice after systemic injection [120].

Instead of injecting naked virions, using cells as delivery vehicles could hide the viral antigen from antibodies and complements. This so-called “Trojan horse” strategy involved infecting the body’s cells *in vitro* and administering these cells back systemically, which would then carry the oncolytic virus to the tumor environment. Cells that have been tested include mesenchymal stem cells [157–159], monocytes [160], outgrowth endothelial cells [160], tumor cells [161–163], T cells [164–166], and dendritic cells (DCs) [165]. Ong *et al*. [167] showed that MV-infected T cells could facilitate tumoral delivery in low, but not high antibody concentration. Power *et al*. [168] tested a number of carrier cells including solid tumor and leukemic cells, and demonstrated that the efficacy of oncolytic vesicular stomatitis virus (VSV) was significantly improved compared to naked virion injection. Interestingly, Zhu *et al*. [169] demonstrated that mice pre-immunized with HSV exhibited reduced growth of S-180 tumor after intratumoral treatment with HSV. PBMCs from seropositive mice showed greater cytotoxicity *in vitro* compared to naïve mice, with higher IFN-γ induction. It is not known if this also applies to intravenous virus delivery or to other oncolytic viral species. Whilst the cell carrier approach has yielded promising data *in vivo*, numerous issues must be considered before clinical application, including the best cell type to use, ease of infection, tumor-targeting capabilities, protection of virus from the host immune response, virus delivery, and tumorigenicity. Recently Kangasniemi *et al*. [170] have demonstrated that silica gel-encapsulated Ads allowed for extended release of the viruses and slightly delayed the development of anti-Ad antibodies. This method has anti-tumoral activity, but comparison with other methods of administration was not performed.

After intravenous delivery the liver, part of the reticuloendothelial system, is the predominant site of Ad5 sequestration with significant hepatocyte transduction [171,172]. Ad5 is known to cause liver toxicity, and its use has raised some concerns after the death of Jesse Gelsinger in 1999 from Ad5-based gene therapy injected directly into the hepatic artery [173]. A landmark study by Waddington *et al*. [174] showed that liver transduction is mediated by interaction of the adenoviral hexon protein with the blood coagulation factor X. This provides a further rationale for the development of other Ad serotypes for oncolytic therapy, such as Ad11 and Ad35 as they bind weakly to factor X compared to Ad5 [118] or other Ad5 chimera. In CD46 transgenic mice, Ad11 persisted much longer in the circulation after intravenous delivery compared to Ad5 together with the absence of liver transduction [120,122]. As for Ad5, ways to reduce liver uptake include recent experiments performed by Barry *et al*. They studied the effect of Kupffer cell depletion (by pre-dosing mice with non-replicating Ad5) and warfarin treatment (to inhibit vitamin K-dependent coagulation factors) and found that this approach significantly increased the anti-tumoral effect of systemically delivered oncolytic Ad5 in nude mice [175]. Good results have also been demonstrated by coating Ad5 with high molecular weight polyethylene glycol [176] or by genetic modification of the hexon protein to ablate blood factor binding [177] for liver detargeting.

A plethora of immunostimulatory genes have been inserted into the genome of oncolytic viruses with the aim of stimulating effective anti-tumoral immune responses. Recent examples include the heat shock proteins [179,180], chemokine (C-C motif) ligand 5 (CCL5) [181], IFN [182], granulocyte macrophage colony-stimulating factor (GM-CSF) [183–185], IL-12 [186], IL-18 [81,82], and IL-24 [187,188]. Vaccinia virus normally expresses a number of type I IFN-inhibiting proteins to counteract the cellular IFN anti-viral response. Because cancer cells frequently have an inactivated IFN pathway, anti-IFN gene-deleted vaccinia could selectively replicate in these cells. Kirn *et al*. [189] utilized this mutant and inserted a gene that encodes IFN-β (which itself has anti-proliferative, anti-angiogenic, and immunomodulatory anti-tumoral effects), and demonstrated enhanced tumor selectivity and potency *in vivo*. Shashkova *et al*. [190] used a four-pronged approach by co-infecting cancer cells with a replicating oncolytic Ad with ADP overexpression and IFN-α expression, given together with a non-replicating virus encoding the gene for TRAIL, with impressive results. The currently used oncolytic MVs were derived from the attenuated Edmonston tag (Edmtag) strain. Significantly, they lack antagonizing activity against the host anti-viral IFN immune response, thus inhibiting virus spread. Recombinant MV encoding the measles phosphoprotein (*P*) gene product from wild-type MV, an IFN antagonist, has been found to exhibit reduced IFN sensitivity and better oncolytic potency *in vivo* [191]. A recombinant VSV vector which expresses a gene from human CMV has been found to have increased anti-tumoral activity *in vivo* [192]. The expressed protein inhibited the natural killer (NK) cell-activating ligand CD155, resulting in decreased accumulation of NK and NKT cells at the infected tumor site and elevated virus replication.

Antigen-specific activation and proliferation of lymphocytes are regulated by interaction of the peptide-antigen-major histocompatibility complex (MHC) with the T cell receptor, as well as both positive and negative signals from co-stimulatory molecules expressed on antigen-presenting cells (APCs). The most important of the APCs are the DCs. DCs are capable of capturing antigens secreted or shed by tumor cells and upon maturation, present the peptides to T cells. Endo *et al*. [193] showed that virus replication led to the production of uric acid in cancer cells, which stimulated DCs to produce IFN-γ and IL-12. IFN-γ subsequently induced the expression of the proteosome activator PA28, which functions to generate tumor antigenic peptides required for MHC class I presentation, resulting in the induction of cytotoxic T lymphocytes (CTLs) against tumor cells. Lapteva *et al*. [194] and Ramakrishna *et al*. [195] demonstrated that increased DC migration and maturation by oncolytic Ad encoding β-defensin-2 or macrophage inflammatory protein 1α (MIP-1α) and Fms-like tyrosine kinase-3 ligand (Flt3L) significantly enhanced anti-tumoral immune responses. Chuang *et al*. [196] used another approach whereby tumor-bearing mice were first primed with DNA encoding a highly immunogenic foreign antigen ovalbumin (OVA), followed by intratumoral injection of vaccinia virus encoding the same antigen. The DNA vaccination served to generate OVA-specific CTLs against infected cancer cells, and the virus resulted in further oncolysis. A study by Diaz *et al*. [135] revealed that depletion of regulatory T cells reduced the efficacy of oncolytic VSV, due to the relief of anti-viral immune response suppression. Anti-tumoral immune activity could be improved by adoptive T cell transfer or incorporation of tumor-associated antigen into the virus. Huang *et al*. [197] utilized an oncolytic Ad armed with IL-12 and 4-1BB ligand, and demonstrated impressive results in mice bearing B16-F10 melanoma tumors. Amongst other functions, 4-1BB ligand (expressed on DCs) enhances T cell proliferation and IL-12 promotes their differentiation. The anti-tumoral effect was even greater when the virus was given together with DCs.

The *E3* region of the adenoviral genome is divided into *E3A* (encodes the 12.5K, 6.7K, gp19K and 11.6K proteins) and *E3B* (10.4K, 14.5K and 14.7K proteins) and is involved in immune response evasion and virus release from cells. Because it is dispensable, this region is frequently deleted in many adenoviral mutants to provide more space for therapeutic gene insertion, although recent work has suggested that transgene expression was higher if gene was inserted at regions other than *E3*, such as *L3* [198]. Deletion of the whole *E3B* region, however, could attenuate the virus oncolytic potency by increasing macrophage infiltration and expression of TNF and IFN-γ [51,133]. Potency could be restored by selective deletion of *E3 gp19K* whilst retaining other *E3* regions [133,199]. In addition to the inhibition of NK cell activation [200], gp19K is an endoplasmic reticulum membrane glycoprotein that inhibits the transport of MHC class I to the cell surface and delays its expression to avoid killing by CTLs [201,202]. CTL evasion is common in tumor cells and therefore the function of gp19k is redundant in these cells. Deletion of this gene, however, would ensure normal cells infected with this virus are eradicated, and in effect this confines virus replication to tumor cells.

## Conclusions

The field of oncolytic virotherapy is expanding and viruses continue to hold promise as effective treatments in combination with chemotherapy or other therapeutic modalities. As continuing work is being done to improve the currently available oncolytic viruses, novel viral species are also emerging and worth exploring, for example the porcine Seneca Valley virus [203], myxoma virus [204], Sindbis virus [205], and Semliki Forest virus [206]. Viruses have unique properties in comparison to small molecular drugs. They can replicate and spread in addition to carry anti-tumoral therapeutic genes. However, during the course of evolution the human body has developed ways to overcome infection and this has imposed a significant barrier towards achieving maximum therapeutic efficacy of oncolytic viruses. Recent advances in our understanding of tumor biology and virology have helped to overcome some of these hurdles, and different groups have successfully targeted features that varied from virus delivery to altering the host immune response. It is hoped that this collective effort will finally pave way for the development of effective and safe viruses for cancer therapy.
